# Klf8 regulates left-right asymmetric patterning through modulation of Kupffer’s vesicle morphogenesis and *spaw* expression

**DOI:** 10.1186/s12929-017-0351-y

**Published:** 2017-07-17

**Authors:** Che-Yi Lin, Ming-Yuan Tsai, Yu-Hsiu Liu, Yu-Fen Lu, Yi-Chung Chen, Yun-Ren Lai, Hsin-Chi Liao, Huang-Wei Lien, Chung-Hsiang Yang, Chang-Jen Huang, Sheng-Ping L. Hwang

**Affiliations:** 1 Department of Bioscience and Biotechnology, National Taiwan Ocean University, Keelung, Taiwan; 2 Graduate Institute of Life Sciences, National Defence Medical Center, National Defence University, Neihu, Taipei, Taiwan; 3 Department of Life Science, National Taiwan University, Taipei, Taiwan; 4 Institute of Cellular and Organismic Biology, Academia Sinica, Nankang, Taipei, 11529 Taiwan; 5 Institute of Biological Chemistry, Academia Sinica, Taipei, Taiwan; 6 Present address: Institute of Cellular and Organismic Biology, Academia Sinica, Taipei, Taiwan

**Keywords:** Zebrafish, Klf8, Spaw, L-R patterning, Kupffer’s vesicle

## Abstract

**Background:**

Although vertebrates are bilaterally symmetric organisms, their internal organs are distributed asymmetrically along a left-right axis. Disruption of left-right axis asymmetric patterning often occurs in human genetic disorders. In zebrafish embryos, Kupffer’s vesicle, like the mouse node, breaks symmetry by inducing asymmetric expression of the *Nodal*-related gene, *spaw*, in the left lateral plate mesoderm (LPM). Spaw then stimulates transcription of itself and downstream genes, including *lft1*, *lft2*, and *pitx2*, specifically in the left side of the diencephalon, heart and LPM. This developmental step is essential to establish subsequent asymmetric organ positioning. In this study, we evaluated the role of *krüppel-like factor 8* (*klf8*) in regulating left-right asymmetric patterning in zebrafish embryos.

**Methods:**

Zebrafish *klf8* expression was disrupted by both morpholino antisense oligomer-mediated knockdown and a CRISPR-Cas9 system. Whole-mount in situ hybridization was conducted to evaluate gene expression patterns of Nodal signalling components and the positions of heart and visceral organs. Dorsal forerunner cell number was evaluated in *Tg(sox17:gfp)* embryos and the length and number of cilia in Kupffer’s vesicle were analyzed by immunocytochemistry using an acetylated tubulin antibody.

**Results:**

Heart jogging, looping and visceral organ positioning were all defective in zebrafish *klf8* morphants. At the 18–22 s stages, *klf8* morphants showed reduced expression of genes encoding Nodal signalling components (*spaw*, *lft1*, *lft2*, and *pitx2*) in the left LPM, diencephalon, and heart. Co-injection of *klf8* mRNA with *klf8* morpholino partially rescued *spaw* expression. Furthermore, *klf8* but not *klf8△zf* overexpressing embryos showed dysregulated bilateral expression of Nodal signalling components at late somite stages. At the 10s stage, *klf8* morphants exhibited reductions in length and number of cilia in Kupffer’s vesicle, while at 75% epiboly, fewer dorsal forerunner cells were observed. Interestingly, *klf8* mutant embryos, generated by a CRISPR-Cas9 system, showed bilateral *spaw* expression in the LPM at late somite stages. This observation may be partly attributed to compensatory upregulation of *klf12b*, because *klf12b* knockdown reduced the percentage of *klf8* mutants exhibiting bilateral *spaw* expression.

**Conclusions:**

Our results demonstrate that zebrafish Klf8 regulates left-right asymmetric patterning by modulating both Kupffer’s vesicle morphogenesis and *spaw* expression in the left LPM.

**Electronic supplementary material:**

The online version of this article (doi:10.1186/s12929-017-0351-y) contains supplementary material, which is available to authorized users.

## Background

Despite the outward appearance of bilateral symmetry in vertebrates, internal organs exhibit substantial left-right asymmetry. In humans, genetic disorders that affect left-right asymmetric patterning may result in organ heterotaxy [1], complex congenital heart disease, and asplenia/polysplenia [2]. In order to study the various processes that establish left-right asymmetry in a laboratory setting, several vertebrates, including mice and zebrafish, have been utilized. Largely based on these animal studies, the major developmental processes which establish asymmetry are known to include: symmetry-breaking in the node, the transfer of asymmetric *Nodal* expression from the node to the left lateral plate mesoderm (LPM), asymmetric expression of *Nodal* and downstream genes in the left LPM, and the completion of left-right asymmetric organ morphogenesis [3, 4].

Clockwise rotation of nodal cilia creates a directional nodal flow, which is responsible for the preferential activation of *Nodal* expression on the left side of the embryo [5]. Not surprisingly, mutations in genes involved in ciliogenesis [6] or its regulation [7, 8] have been found to disrupt normal left-right patterning. Leftward nodal flow generates an initial accumulation of NODAL protein on the left side of the embryo. Subsequently, self-enhancement and lateral-inhibition systems involving NODAL, LEFTY1 and LEFTY2 reinforce the asymmetric distribution and restrict *Nodal* gene expression to the left side of the organism [9].

Nodal signalling is initiated by the binding of NODAL to the ACTIVIN receptor and EGF-CFC co-receptor, which results in the formation of an intracellular regulatory complex. This complex consists of phosphorylated SMAD2, SMAD4 and FoxH1, and directly activates target gene transcription [10]. Left-side specific enhancers (ASEs) with FoxH1 binding motifs are present in the murine *Nodal* and *Lefty2* genes [11]. Thus, NODAL amplifies its own expression in the left LPM via SMADs/FoxH1 interaction with the ASE. Simultaneously, NODAL induces *Lefty2* expression, which inhibits low-level NODAL signalling, and thereby restricts *Nodal* expression to the left LPM. This asymmetric NODAL activation induces expression of *Pitx2* in the left LPM, via its ASE. PITX2 is a homeodomain transcription factor implicated in left-right asymmetric organ morphogenesis [12], and loss of *Pitx2* expression has been shown to affect the asymmetric distribution of internal organs in several vertebrates [13].

In zebrafish embryos, Kupffer’s vesicle (KV) performs a similar role to the mouse node in initiating left-right asymmetric patterning [14]. KV is derived from dorsal forerunner cells (DFCs), which are formed via a Nodal signalling-dependent ingression of surface enveloping layer cells from the dorsal blastoderm margin. This ingression occurs at the blastula stage, when embryonic epiboly initiates [15]. DFCs then migrate toward the vegetal pole and organize into multiple rosette-like, epithelial structures at the end of gastrulation. These epithelial rosettes then merge into a single epithelial rosette and differentiate into the ciliated KV, with the vesicle lumen arising from apical membrane expansion during early somite stages. Tilted cilia are positioned with the basal body at the posterior pole of DFCs, and these motile cilia are asymmetrically distributed along the anterior-posterior axis of KV. Furthermore, cilia asymmetry is established by the Rho kinase, Rock2b, and is essential to generate an anti-clockwise swirling flow that commences asymmetric Nodal signalling [16–18]. In addition to *rock2b*, deficiencies in several genes involved in the movement, formation or positioning of cilia, such as *dnah9*, *ift88*, and *vangl2*, have been shown to disrupt normal left-right patterning [14, 19, 20].

Three *nodal*-related genes, namely *ndr2* (*cyc*), *ndr1* (*sqt*), and *southpaw* (*spaw*), have been identified in zebrafish [10]. Among these genes, *spaw* exhibits the earliest expression in the left-side of the LPM, and stimulation of its own transcription during somitogenesis shifts its expression domain from the posterior to the anterior left LPM [21, 22]. Furthermore, morpholino knockdown of *spaw* decreases expression of genes encoding Nodal signalling components, including *spaw*, *lefty1* (*lft1*), *lefty2* (*lft2*), and *pitx2*, in the left LPM [22], affecting the left-right asymmetric distribution of heart, pancreas, and diencephalon. Together, these studies demonstrate the essential role of Spaw, and underscore its relevance as a NODAL homolog in establishing left-right asymmetry of teleosts [22, 23].

Similar to mouse embryos, different repressors of the Nodal signalling pathway have been reported to modulate the induction or maintenance of asymmetric Nodal signalling in teleosts. At the 4–6 s stages, *spaw* is expressed bilaterally in KV, while *charon* is expressed in a region adjacent to KV, where it antagonizes Spaw activity and contributes to biased *spaw* expression in the left LPM [24]. Furthermore, repression of Spaw activity in the right LPM or cardiac field by Lft1 or Lft2 is also essential in the establishment of left-right patterning. Notably, *lft1* expression in the notochord is induced via binding of BMP4 with BMP receptor 1aa at the early segmentation stage, while *lft2* expression in the left cardiac field is activated by Spaw in the anterior LPM [25–27]. Despite this detailed knowledge about Spaw repressor proteins, it is still unknown whether asymmetric *spaw* expression in the left LPM can be regulated by transcription factors.

Krüppel-like factor 8 (KLF8) is a member of the KLF family of transcription factors [28, 29], and participates in a broad range of developmental processes. KLF proteins contain C-terminal zinc finger DNA binding motifs, and distinct N-terminal regulatory elements. KLF8, like KLF3 and KLF12, possesses a regulatory domain that interacts with C-terminal binding protein (CtBP) [30]. Interaction of KLF8 with the co-repressor CtBP inhibits embryonic *Gamma-Globin* gene expression [31, 32], a role confirmed in *klf8*-deficient mice [33]. KLF8 also functions as a mediator of focal adhesion kinase to activate *cyclin D1* expression, modulating cell cycle progression [34]. Recently, we used morpholino knockdown and rescue experiments to show that zebrafish Klf8 has a novel function in cerebellar development. In this context, Klf8 modulates the expression of *p53* and *met* to maintain *ptf1a*-expressing neuronal progenitors, which are required for proper development of cerebellar Purkinje and granule cells [35]. In addition, we noted that *klf8* morphants often exhibited a no-loop heart at 48 h post fertilization (hpf).

In this study, we demonstrate that zebrafish Klf8 plays an additional role in regulating left-right asymmetric patterning. Heart jogging, looping and visceral organ positioning were defective in *klf8* morphants. At 18–22 s stages, expression levels of *spaw*, *lft1*, *lft2*, and *pitx2* were decreased or eliminated in the left LPM, diencephalon, and heart of the majority of *klf8* morphants. In contrast, *klf8* overexpression resulted in bilateral expression of *spaw* and its downstream target genes in these tissues. Both dorsal forerunner cell number, and the length and number of cilia in KV were also affected in *klf8* morphants. However, *klf8* CRISPR-Cas mutant embryos showed bilateral *spaw* expression in the LPM, which may have been partly due to compensatory upregulation of *klf12b*.

## Methods

### Ethics approval

All animal procedures were approved by the Institutional Animal Care and Use Committee of Academia Sinica (Protocol ID: 15–12-918).

### Zebrafish maintenance and staging

Adult AB zebrafish, *Tg(sox17:gfp)* ^*s870/+*^ and *klf8* mutants (*klf8* ^*d25*^, *klf8* ^*i17*^), generated by a CRISPR-Cas9 system were maintained in high density, self-circulation systems (Aqua Blue), or 20 L aquaria supplied with filtered fresh water and aeration under a photo period of 14 h light and 10 h dark. Embryos were maintained at 28.5 °C, and morphological criteria were defined as described [36].

### Plasmid construction, morpholino and mRNA injection

The full-length *klf8* coding sequence or *klf8* lacking zinc finger domain (*klf8△zf*) was cloned into the T7TS vector, and used as template to synthesize capped mRNA with the mMESSAGE mMACHINE T7 Kit (Ambion). Previously published morpholinos (MOs) were used [35], including two MOs that prevent Klf8 protein translation: *klf8*-MO1^atg^ (2.2 ng) and *klf8*-MO2^atg^ (1.9 ng), two MOs that prevent *klf8* mRNA splicing: MO^DO^ (0.73 ng) and MO^AC^ (0.73 ng), and one control MO: *klf8*-4 mm MO1 (2.2 ng). An additional MO to prevent *klf8* mRNA splicing: MO^DO2^ (0.73 ng; 5′- TGGGTCACATCCATCTCACCTGATC -3′; targets the donor site of exon 3) was used. A 1.5-fold greater dosage of *P53* MO^sp^ [37] was injected, as compared to the co-injected *klf8*-MOs. To verify upregulation of *klf12b* in homozygous *klf8* ^*d25*^ mutant F6 embryos, *klf12b* MO (5 or 10 ng) was used. *klf12b* MO (5′-ATTCCGTCTAGCATTAACATCCTGT-3′), which is complementary to 20 bases of the coding region including the ATG start codon (underlined) and five bases of the 5′ untranslated region, was used. The indicated MO or mRNA was microinjected into one- or two-cell zygotes using a Nanoject II automatic injector (Drummond).

### Whole mount in situ hybridization, whole mount immunohistochemistry, and photography

Whole mount in situ hybridization was performed on embryos treated with 0.003% phenylthiocarbamide, using digoxigenin-antisense RNA probes and alkaline phosphatase-conjugated anti-digoxigenin antibody. Various templates derived from pGEMT or pGEMT-Easy vectors were linearized, and the following antisense RNA probes were generated (restriction site and promoter in parentheses): *bmp2b* (BamHI/T7), *charon* (BamHI /Sp6), *myl7* (NcoI /SP6), *gata5* (SacII/SP6), *gata 6* (SalI/T7), *lft1* (SalI/T7), *lft2* (HindIII/SP6), *ntl* (XhoI/T7), *oep* (NcoI/SP6), *pitx2c* (EcoRI/T7), and *spaw* (SpeI/T7). To produce the *dvr1* antisense RNA probe, PCR product that was generated using M13 forward and M13 reverse primers was used as a template and transcribed by T7 RNA polymerase.

To detect changes in KV cilia, one- or two-cell zygotes of *Tg(sox17:gfp)* were microinjected with different *klf8*-MOs. The 10s stage embryos were fixed in 4% paraformaldehyde for 2 h at room temperature (RT) and dehydrated in methanol at −20 °C. After dehydration, the embryos were permeabilized using acetone at −20 °C and washed by Phosphate-buffered saline with tween 20 (PBST) followed by blocking in 5% serum. The embryos were incubated with anti-acetylated tubulin antibody (1:250, Sigma-Aldrich) for 2 h, at RT, followed by mouse Alexa Fluor 568 for 1 h, at RT (1:250, Invitrogen).

To investigate DFC number alteration, one- or two-cell zygotes of *Tg(sox17:gfp)* were microinjected with different *klf8*-MOs. 75% embryos were fixed in 4% paraformaldehyde at 4 °C overnight. After dehydration, the embryos were permeabilized using acetone at −20 °C for 7 min and treated with 0.15 M Tris-HCl, pH 9.0 at 70 °C for 15 min. The embryos were washed with PBST followed by blocking in 1% blocking solution (Roche) at RT for 2 h. The embryos were incubated with anti-GFP antibody (1: 200) at 4 °C overnight, followed by rabbit Alexa Fluor 488 (1:200, Invitrogen) incubation for 1 h, at RT. Nuclei were then stained with Hoechst 33,341 (1:1000 in PBST, Invitrogen).

Brightfield embryo images were taken using an AxioCam HRC camera mounted on a Zeiss Imager M1 microscope. Fluorescent images were taken using a Leica TCS-SP5-MP confocal microscope.

### Generation of *klf8* mutants using CRISPR-Cas9 system

*klf8* mutants were generated using a CRISPR-Cas9 system targeting exon 2. sgRNA was designed by ZGENEBIO BIOTECH INC. (Taipei, Taiwan) and DNA template was amplified from pZGB plasmids containing *klf8* sgRNA. PCR was conducted using forward (5′-ACACAGGAAACAGCTATGACCATG-3′) and reverse (5′-GATCCG CACCGACTCGGTGCCACTTT-3′) primers, and *klf8* sgRNA were synthesized using the MEGAshortscript T7 Transcription Kit (Ambion, Austin, TX, USA). *klf8* sgRNA (86.3 pg) and capped *nls-zCas9-nls* mRNA (34.5 pg, Addgene) [38] were co-injected into one-cell zygotes. Genomic DNA was isolated from pools of 10 embryos at 24 hpf. PCR was conducted using forward (5′- TCTTTCTACTCCTCCCCCAACTAA-3′) and reverse (5′- CCACACCCCTTTCCAATAACTCTA-3′) primers and amplified DNA was then digested with T7 endonuclease I to evaluate insertion and deletion efficiency. The rest of the embryos were reared to adulthood. Injected fish were designated as the F0 generation. To detect the DNA sequence alterations induced by *klf8* sgRNA, genomic DNA was isolated from clipped tail fin of adult F1 fish, and high resolution melt analysis was performed. PCR was conducted in a 20 μL reaction comprising 8–12 ng genomic DNA, 3.5 mM MgCl_2_, 1× Master Mix containing Taq DNA polymerase, dNTP mix and LightCycler 480 ResoLight dye, and 5 pmol each of forward (5′- ATCTCAGAACTCGGGTCACTTTTT-3′) and reverse (5′- CCACCATACACTCCACCTCCTC-3′) primers. The PCR conditions were 95 °C for 300 s, 1 cycle for pre-incubation, 95 °C for 10 s, 65 °C to 53 °C with a 0.5 °C gradient decline for 10 s, and 72 °C for 10 s, for 70 cycles of amplification, and 95 °C for 60 s, 40 °C for 60 s, 65 °C to 95 °C for 1 s melting interval for high resolution melting. DNA sequencing was conducted to confirm F1 adult fish with induced DNA sequence alterations. Two *klf8* F1 mutants including *klf8* ^*d25*^ with a 25 bp deletion and *klf8* ^*i17*^ with a 17 bp insertion in the exon 2 *klf8* sgRNA target site were crossed with wild type fish to produce the F2 generation. Subsequently, homozygous F4 *klf8* ^*d25*^ and *klf8* ^*i17*^ mutants were generated by intercrossing of respective heterozygous F3 mutants and maintained to the F5 generation.

### Reverse transcription PCR (RT-PCR) and reverse transcription quantitative real-time PCR (RT-qPCR)

RT-PCR was used to evaluate the efficacy of sp.-MOs. cDNA from 24 hpf, forward primer (5′- ATCAAGCCGGAGCCAGAGGAGGTG-3′) and reverse primer (5′-GCCGTCGGTGAAGTGCCAGGTG-3′) were used. RT-qPCR [39] was used to compare expression levels of *klf3*, *klf12a* or *klf12b* in wild type and homozygous F5 *klf8* ^*d25*^ and *klf8* ^*i17*^ mutant embryos. cDNA was generated by a GoScript Reverse transcription system (Promega) using total RNA isolated from 10 to 12 s stage wild type or two homozygous *klf8* ^*d25*^ and *klf8* ^*i17*^ mutant embryos. RT-qPCR was conducted in a 10 μL reaction containing 1× LightCycler 480 SYBR Green I Mix (Roche), respective primer pairs (5 μg) and 1/10 cDNA from wild type or mutant embryos. PCR conditions were 95 °C for 10 min, then 55 cycles of, 95 °C for 10 s, 60 °C for 10 s, and 72 °C for 10 s, followed by a 4 °C pause. Primer pairs for *klf3* were forward (5′-TATCCAAGTGGACATTACTGTGGG-3′) and reverse (5′-CAGTGGGCAACACAGAACGGCAG-3′). Primer pairs for *klf12a* were forward (5′-GAGCGGGTCTCTTTCTGCCAGTG-3′) and reverse (5′-CAATAAACCGTATGAGGGAAAGGC-3′). Primer pairs for *klf12b* were forward (5′-GGCAATCCCTGCTCCTCAGAAAC-3′) and reverse (5′-CCACATCGTAGACTCCAAAATGCG-3′).

### Quantification of cilia number and length and lumen of Kupffer’s vesicles as well as DFC number

The cilia length and number were quantified using LAS AF and MetaMorph software according to the following steps: (i) merge images with LAS AF for MetaMorph analysis; (ii) “Threshold Image” was set to demarcate cilia and KV cell locations, and the image was converted to grayscale; (iii) from the Arithmetic menu, “Logical AND” was selected, and KV cilia regions were defined; (iv) “Calibrate Distances” was set to define units of length (μm); and (v) the length and number of cilia were quantified by selecting “Integrated Morphometry Analysis” in the Measure menu. The area of KV lumen was quantified using ImageJ software as follows: (i) The merged grayscale images from MetaMorph were loaded in ImageJ; (ii) “Elliptical selections” was selected to demarcate cilia area; (iii) “Set scale” was selected to define units of area (μm^2^); and (iv) the area was determined by selecting “Measure” in the Analyze menu.

In order to evaluate the DFC number, immunofluorescence confocal images of *Tg(sox17:gfp)* 75% epiboly embryos were merged using ImageJ software using the following steps: (i) Images were loaded into ImageJ; (ii) “Images to stack” was selected from the “Stacks” item in the Image menu; (iii) “Stacks Focuser” was selected from the “Stacks” item in the Plugins menu. DFC number was then manually counted from images with merged GFP and nuclei.

### Statistics

Two-tailed Student’s *t*-tests with unequal variance were conducted to compare number of cilia, cilia length, lumen area and DFC. To compare the *klf8* mRNA rescue effect, Fisher’s Exact Test was used. Statistical tests were performed with Excel software. Differences with *p* < 0.05 were considered to be statistically significant.

## Results

### *klf8* morphants display abnormal heart jogging, looping and visceral organ positions

**Fig. 1:**
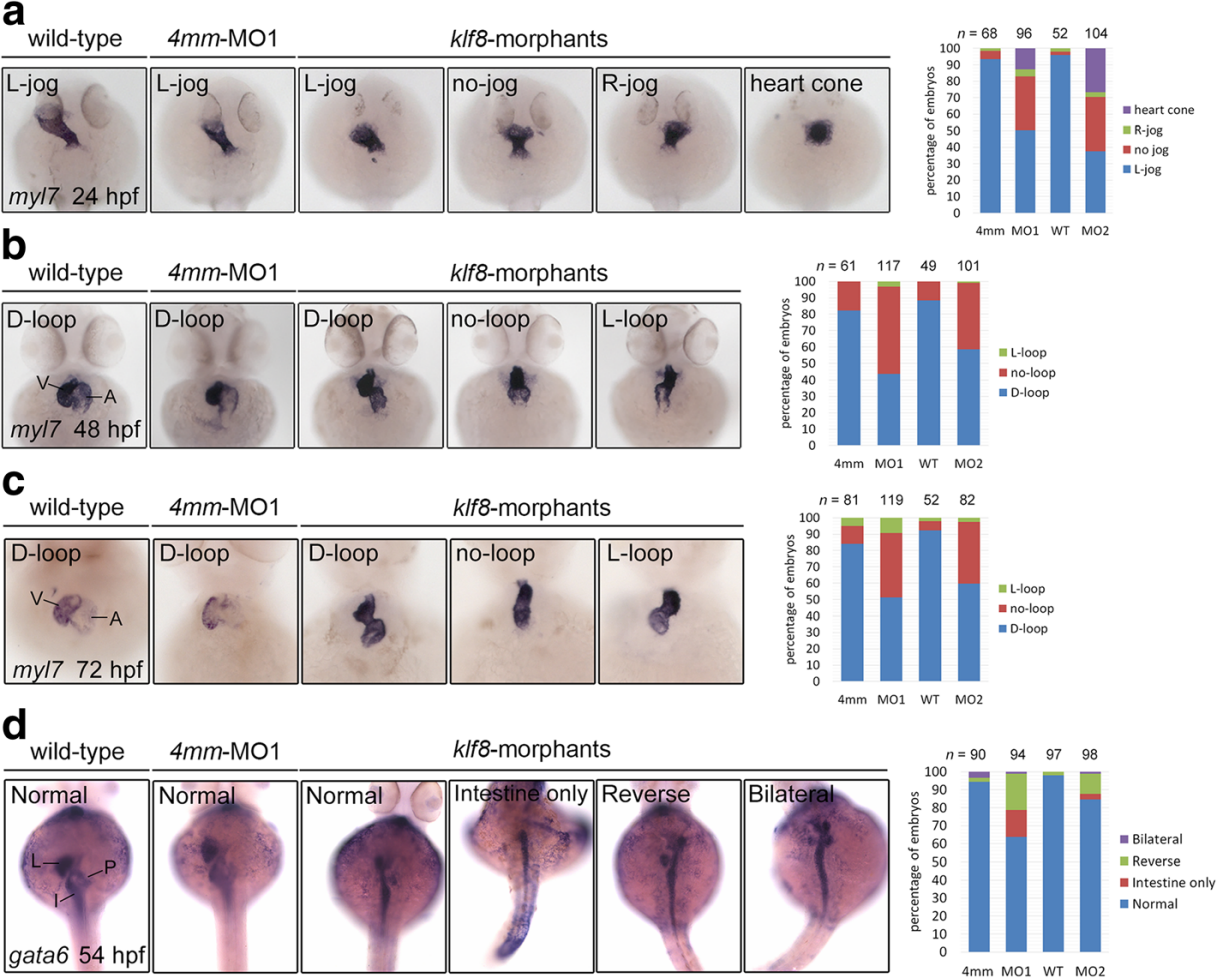
Knockdown of zebrafish *klf8* caused defects in heart jogging and looping, and visceral organ positions. **a** *klf8*-MO1^atg^ or *klf8*-MO2^atg^ -injected embryos stained with *myl7* exhibited left (L)-jog, no-jog or right (R)-jog and were compared to stained wild-type or 4 mm-MO1-injected control embryos at 24 hpf. **b** *myl7* stained embryos injected with *klf8*-MO1^atg^ or *klf8*-MO2^atg^ displayed D-loop, no-loop or L-loop heart and were compared to wild type and control embryos at 48 hpf. **c** *myl7* stained embryos injected with *klf8*-MO1^atg^ or *klf8*-MO2^atg^ displayed D-loop, no-loop or L-loop heart and were compared to wild type and control embryos at 72 hpf. **d** At 54 hpf, *gata6* stained wild type or embryos injected with *klf8*-4 mm MO1, *klf8*-MO1^atg^ or *klf8*-MO2^atg^ exhibited organ positions that were classified as: (Normal) normal positions of liver-left, pancreas-right and intestine with left looping, (Intestine only) only intestine without left looping, (Reverse) reversed position of liver-right, pancreas-left and no looping intestine, or (Bilateral) bilateral extension of liver and pancreas. A, atrium; I, intestine; L, liver; P, pancreas; V, ventricle

During a previous study investigating the role of Klf8 in cerebellar development [35], we noted that *klf8* morphants often exhibited a no-loop heart at 48 hpf. Because cardiac development is asymmetric, this observation suggested that Klf8 may regulate the general process of left-right patterning. Thus we performed *klf8* knockdown experiments with previously validated *klf8*-MOs and systematically evaluated heart morphogenesis by whole-mount in situ hybridization, using a *myl7* antisense RNA probe. A phenotypically normal, L-jog heart tube was readily detected in wild type and *klf8*-4 mm MO1-injected embryos, while embryos injected with *klf8*-MO1^atg^ or *klf8*-MO2^atg^ often showed no-jog (32% for MO1^atg^, 33% for MO2^atg^) or right-jog (4% for MO1^atg^, 3% for MO2^atg^) heart tubes at 24 hpf (Fig. 1a). Consequently, embryos injected with *klf8*-MO1^atg^ or *klf8*-MO2^atg^ frequently developed a no-loop heart (53% for MO1^atg^, 40% for MO2^atg^) as compared to the vast majority of wild type or *klf8*-4 mm MO1-injected embryos showing a D-loop heart at 48 hpf (Fig. 1b). At 72 hpf, a similar percentage of *klf8*-MO1^atg^- or *klf8*-MO2^atg^-injected embryos displayed a no-loop heart (40% for MO1^atg^, 38% for MO2^atg^) as compared to wild type or *klf8*-4 mm MO1-injected embryos, which almost invariably had a D-loop heart (Fig. 1c). Embryos showing a delayed heart cone phenotype were also identified in the *klf8*-MO1^atg^ (13%) or *klf8*-MO2^atg^ (27%) groups at 24 hpf (Fig. 1a). However, this delayed phenotype was not observed in the 48 hpf or 72 hpf time points. By these stages, the delayed morphants caught up developmentally and displayed either D-loop, no-loop or L-loop hearts (Fig. 1b&c). We also examined the position of digestive organs in *klf8* morphants by whole-mount in situ hybridization, using a *gata6* antisense RNA probe. In *klf8*-MO1^atg^- or *klf8*-MO2^atg^-injected embryos, frequent occurrence of organ dysmorphologies were observed. Reversal of liver and pancreas position (20% for MO1^atg^, 11% for MO2^atg^), only intestine development (15% for MO1^atg^, 3% for MO2^atg^), and bilateral liver and pancreas (1% for MO1^atg^, 1% for MO2^atg^) were all frequently detected in *klf8* morphants, but were rare events in wild type or control embryos at 54 hpf (Fig. 1d). These results confirmed that *klf8* loss-of-function affects left-right patterning in zebrafish embryos.

### *klf8* deficiency affects the level and pattern of expression for genes in the Nodal signalling pathway

**Fig. 2:**
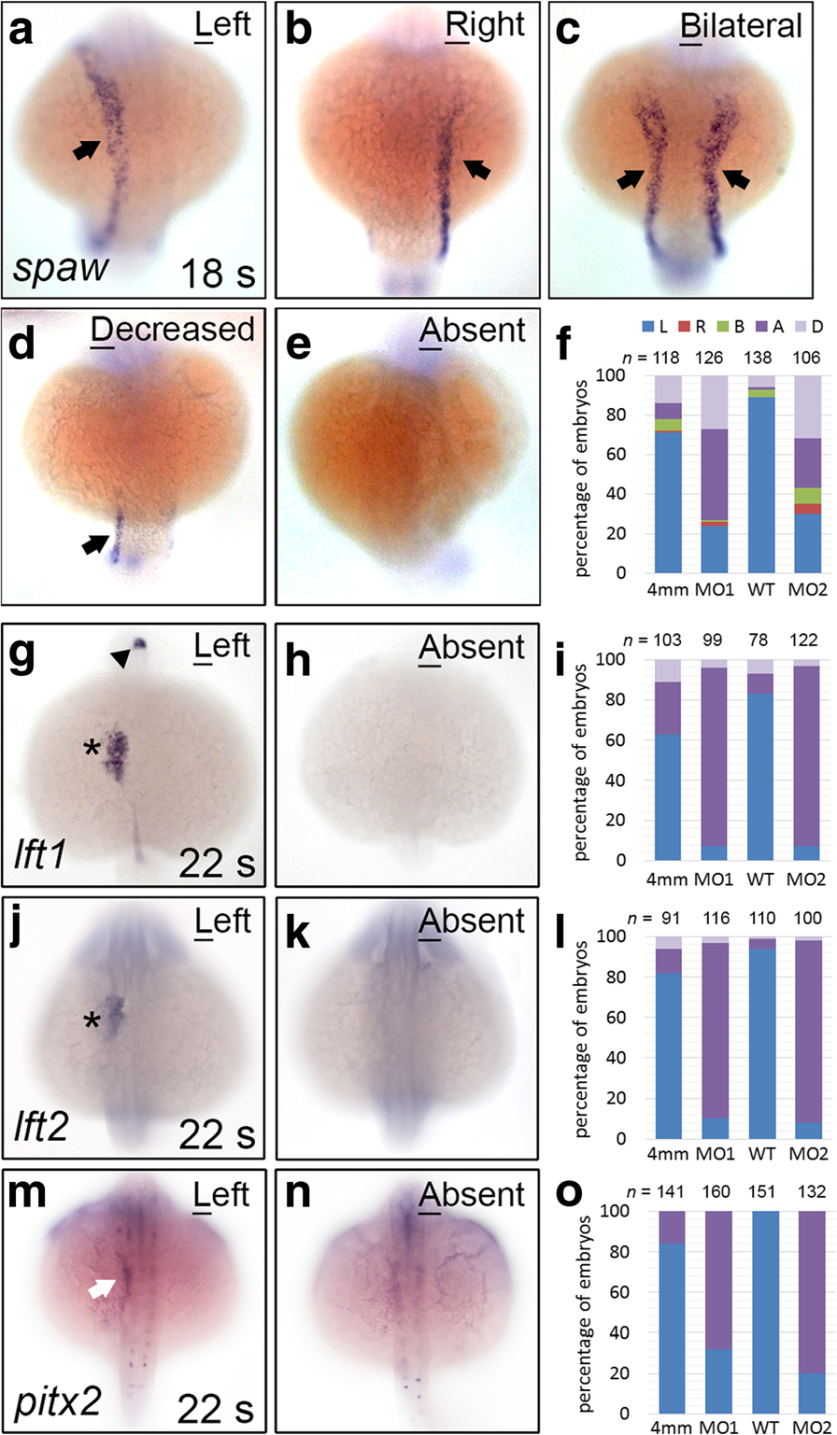
Genes encoding Nodal signalling components exhibited decreased or abolished expression in *klf8* morphants. The majority of *klf8*-MO1^atg^ or *klf8*-MO2^atg^ -injected embryos showed decreased or abolished *spaw* (*arrow*) expression in the left lateral plate mesoderm (LPM) at the 18 s stage (**a-f**). The majority of *klf8* morphants failed to express *lft1* in the left diencephalon (*arrowhead*) or heart (*asterisk*) (**g- i**), *lft2* in the left heart (*asterisk*) (**j-l**), and *pitx2* in the left LPM (*white arrow*) (**m-o**) at the 22 s stage. Dorsal views of embryos are shown. A, absent; B, bilateral; D, decreased; L, left; R, right

Genes encoding Nodal signalling components, including *spaw*, *lft1*, *lft2*, and *pitx2*, were asymmetrically expressed in the left side of the diencephalon, heart, or lateral plate mesoderm (LPM) during the 18–22 s stages in zebrafish embryos (Fig. 2a–m). Disruption in the expression of left-side specific Nodal signalling genes results in organ heterotaxy. We observed that *spaw* expression in the left LPM was either decreased (no expression in the anterior LPM and low expression in the posterior LPM, 27%) or absent (46%) in many *klf8*-MO1^atg^-injected embryos (Fig. 2d–f). A similar effect (32% decreased, 25% absent, 8% bilateral, 5% right) was observed following *klf8*-MO2^atg^ injection (Fig. 2b-f). Consistent with these results, the expression of genes downstream of *spaw* (*lft1*, *lft2*, and *pitx2*) was also absent or decreased in the left diencephalon, heart, and LPM of most *klf8*-MO1^atg^ or *klf8*-MO2^atg^ -injected embryos (Fig. 2h–o). Additionally, we injected three splicing MOs (MO^DO2^, MO^AC^, and MO^Do^) to block splicing of *klf8* mRNA (Additional file 1: Figure S1, A). RT-PCR indicated that splicing of *klf8* mRNA was effectively disrupted in MO^sp-MOs^-injected embryos at 24 hpf (Additional file 1: Figure S1, B). Expression of *spaw* in the left LPM was either decreased (8%) or eliminated (63%) in most 18 s stage embryos injected with MO^sp-MOs^ (Additional file 1: Figure S1, C).

Klf8 was previously shown to repress *p53* expression and induce *met* expression to modulate the development of Purkinje cells and proliferation of granule cells [35]. To confirm that defective left-right patterning did not arise from induction of *p53* due to *klf8* deficiency, we analysed heart looping and gene expression of Nodal signalling components in embryos that were co-injected with *p53*-MO^sp^, alongside *klf8*-MO1^atg^ or *klf8*-MO2^atg^.

Of the embryos co-injected with *p53*-MO^sp^, together with *klf8*-MO1^atg^ or *klf8*-MO2^atg^, 35–39% exhibited a no-loop heart at 72 hpf, similar to embryos injected with *klf8* MOs alone (Additional file 2: Figure S2, A). Likewise, expression levels of *spaw*, *lft1*, *lft2*, and *pitx2* were reduced or eliminated in the left LPM, diencephalon, and heart of high percentages of co-injected embryos during the 18–22 s stages (Additional file 2: Figure S2, B-E). We also found that the expression levels of *gata5* and *oep* (which are known to be expressed in the LPM at the 22 s stage) were unaffected by *klf8* knockdown (Additional file 2: Figure S2, F-M). Together these data clearly indicate that increased p53 expression and apoptosis are not responsible for the decreased expression of genes involved in Nodal signalling.

**Fig. 3:**
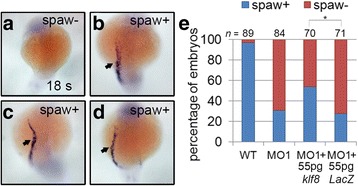
Reduced *spaw* expression in *klf8* morphants was partially rescued by co-injection of *klf8* mRNA. Representative embryos showing *spaw* expression in the left LPM (spaw+) or absent *spaw* expression (spaw-) are shown (**a-d**). Percentages of embryos with asymmetric *spaw* expression or no *spaw* expression are shown with indicated treatments (**e**). Statistical significance was determined by Fisher’s Exact Test. *p < 0.05

To further confirm that the decrease in *spaw* expression is a consequence of *klf8* loss-of-function, we performed rescue experiments by co-injecting embryos with *klf8*-MO1^atg^ and *klf8* mRNA. Approximately 69% of *klf8*-MO1^atg^-injected embryos exhibited eliminated or decreased *spaw* expression in the left LPM at the 18 s stage (Fig. 3a, e). However, this proportion showed a statistically significant reduction to 44% for embryos co-injected with *klf8*-MO1^atg^ and *klf8* mRNA (Fig. 3b-e). Taken together, these results demonstrate that *klf8* loss-of-function causes downregulation of Nodal signalling component genes.

### Overexpression of *klf8* mRNA causes bilateral expression of genes involved in Nodal signalling

**Fig. 4:**
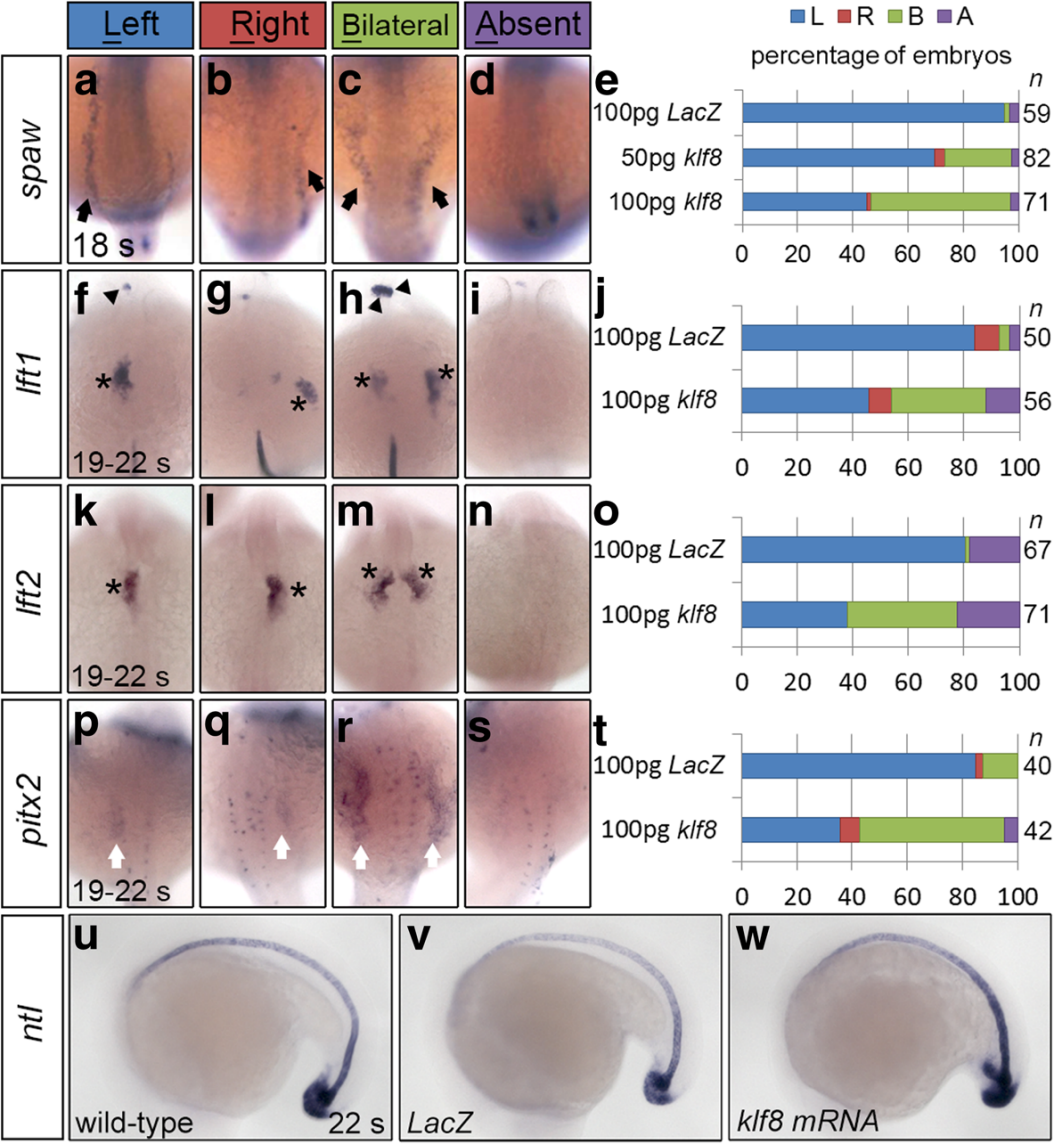
Overexpression of *klf8* mRNA caused bilateral expression of Nodal signalling component genes. Injection of *klf8* mRNA induced bilateral expression of *spaw* in the LPM (*arrow*) at the 18 s stage, in a dose-dependent manner (**a-e**). Embryos injected with *klf8*, but not *LacZ*, exhibited bilateral expression of *lft1* in the diencephalon (*arrowhead*) and heart (*asterisk*; **f-j**), *lft2* in the heart (*asterisk*; **k-o**), and *pitx2* in the LPM (*white arrow*) (**p-t**) at the 19–22 s stage. Expression of *ntl* in the notochord is similar in wild type and embryos injected with *LacZ* or *klf8* mRNA (**u-w**). A, absent; B, bilateral; L, left; R, right

Since *klf8* knockdown reduced expression of genes involved in Nodal signalling, we hypothesized that *klf8* overexpression may have the opposite effect. While the majority of embryos injected with 100 pg of *LacZ* mRNA expressed *spaw* exclusively in the left LPM at the 18 s stage, 24% of embryos injected with 50 pg and 51% of embryos injected with 100 pg of *klf8* mRNA expressed *spaw* bilaterally in the LPM (Fig. 4c, e). Thus, a dose-dependent effect of *klf8* expression was revealed. Moreover, embryos overexpressing *klf8* also frequently exhibited bilateral expression patterns of *lft1, lft2*, and *pitx2* in the diencephalon, heart, and LPM at 19–22 s stages (Fig. 4h–t). On the other hand, *ntl* expression in the notochord was not altered in 22 s stage embryos overexpressing *klf8* as compared to *LacZ*-overexpressing embryos (Fig. 4u-w). In order to investigate whether the zinc finger DNA binding domain of Klf8 is involved in regulating the expression pattern of *spaw* or its downstream genes, we injected mRNA for *klf8* lacking the zinc finger DNA binding domain (*klf8△zf*). We found that injection of 100 pg *klf8△zf* only induced a low percentage of embryos to exhibit bilateral expression of *spaw* (6.3%), *lft1* (3.6%), *lft2* (1.6%) or *pitx2* (11.9%), compared to higher rates of bilateral *spaw* (48.3%), *lft1* (31.6%), *lft2* (37.5%) or *pitx2* (44.3%) expression in *klf8* injected embryos at 18 s or 19–22 s stages (Additional file 3: Figure S3). These results demonstrate that overexpression of *klf8* does not affect the midline structure, but induces ectopic expression of *spaw* and its downstream genes when the Klf8 zinc finger DNA binding domain is intact.

### *klf8* deficiency affects morphogenesis of Kupffer’s vesicle and asymmetric *charon* expression

**Fig. 5:**
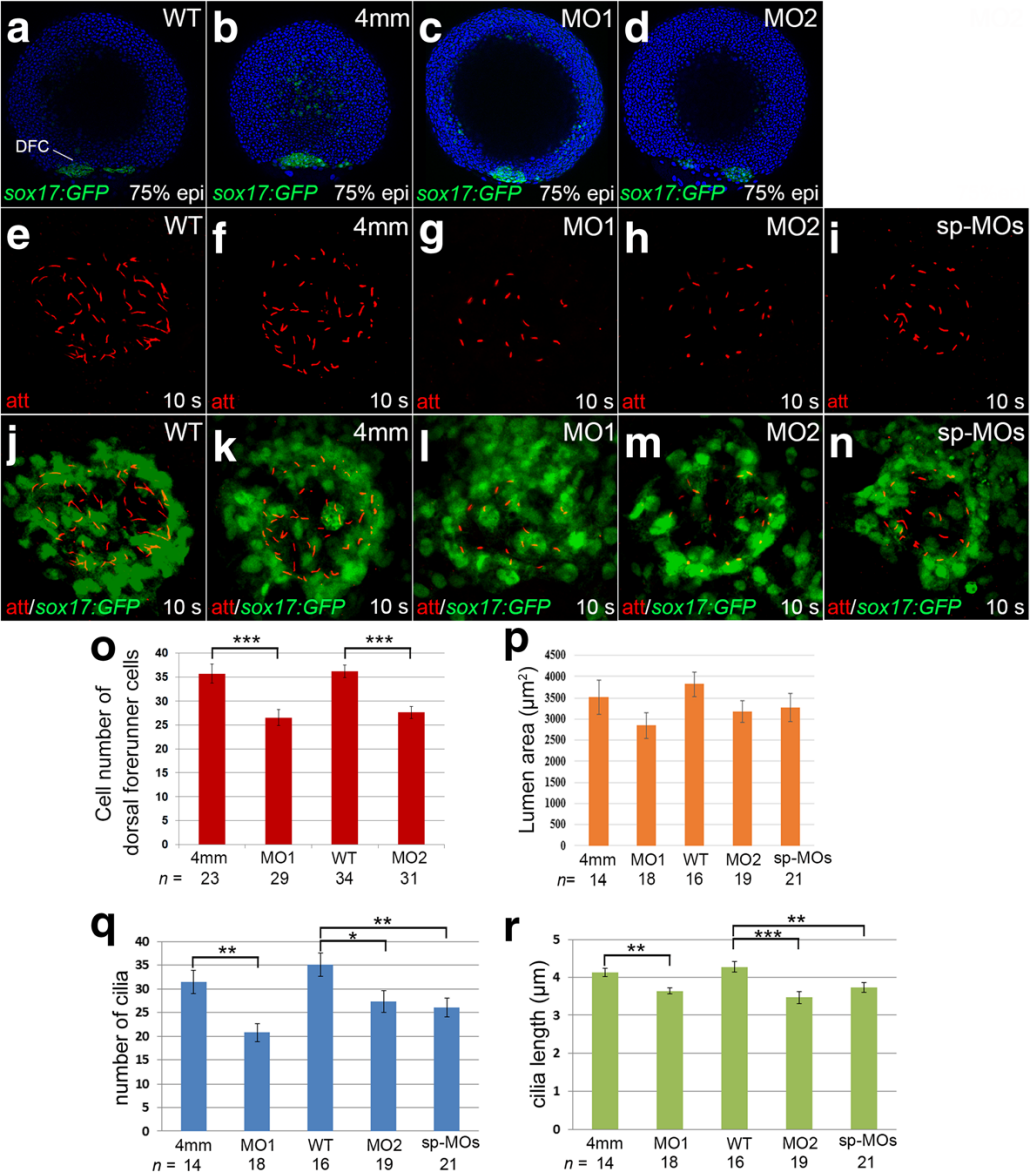
Knockdown of *klf8* affected dorsal forerunner cell number, KV cilia number and length. Cell number of dorsal forerunner cells (DFCs) was affected in *klf8-*MO1^atg^- or *klf8*-MO2^atg^ -injected embryos as compared to *klf8*-4 mm MO1-injected *Tg(sox17:gfp)* control embryos (4 mm) at 75% epiboly (**a-d**). Images of KV cilia stained with acetylated tubulin antibody in *klf8* morphants (**g-i**) and control embryos (**e, f**) at 10s stage (**e-i**). Images of acetylated tubulin stained KV cilia and GFP stained DFCs in *Tg(sox17:gfp)* embryos injected with different *klf8* MOs (**l-n**), *klf8*-4 mm MO1 (**k**) or wild type (**j**) embryos at 10s stage (**j-n**). DFC number (**o**), KV cilia number (**q**), length (**r**) but not lumen area (**p**) were affected in *klf8* morphants as compared to control embryos. Statistical significance was determined by Student’s *t*-test. *p < 0.05, **p < 0.01, ***p < 0.001. *Error bars* indicate standard deviation

Since asymmetric flow, generated by rotation of cilia within KV, is essential to initiate left-right asymmetric patterning, and KV is derived from dorsal forerunner cells (DFCs), we then investigated whether *klf8* knockdown affected cilia or DFC number during KV morphogenesis. Individual *klf8*-MOs or control MO were microinjected into one- or two-cell *Tg(sox17:gfp)* zygotes, and immunofluorescence was conducted using anti-GFP antibody. We found that the number of DFCs at the dorsal margin was significantly reduced in *klf8*-MO1^atg^- (average of 26.6 DFCs) or *klf8*-MO2^atg^- (average of 27.6 DFCs) injected *Tg(sox17:gfp)* embryos as compared to wild type (average of 36.2 DFCs) or *klf8*-4 mm MO1- (average of 35.7 DFCs) injected embryos at 75% epiboly (Fig. 5a-d, o). Cilia were then detected by immunofluorescence staining of 10s stage embryos using an anti-acetylated tubulin antibody. KV lumen size was smaller, but not significantly so, in *Tg(sox17:gfp)* embryos injected with *klf8*-MO1^atg^, *klf8*-MO2^atg^, or MO^sp-MOs^ as compared to wild type or *klf8*-4 mm MO1-injected control embryos at 10s stage (Fig. 5e-n, p). Significantly reduced number and length of KV cilia were detected in embryos injected with different *klf8*-MOs as compared to wild type and control embryos (Fig. 5q, r). Since asymmetric *charon* expression on the right side of the KV was influenced by strength and direction of KV flow [40], we also examined whether *klf8* knockdown affected asymmetric *charon* expression around KV. The majority (61% for MO1^atg^, 57% for MO2^atg^) of embryos injected with different *klf8*-MOs revealed symmetric *charon* expression with reduced expression area around KV as compared to wild type and control embryos at the 10s stage (Additional file 4: Figure S4). These results indicate that KV morphogenesis, cilia length, cilia number and asymmetric *charon* expression were affected in *klf8* knockdown embryos.

### Generation of *klf8* mutant by a CRISPR-Cas9 system

**Fig. 6:**
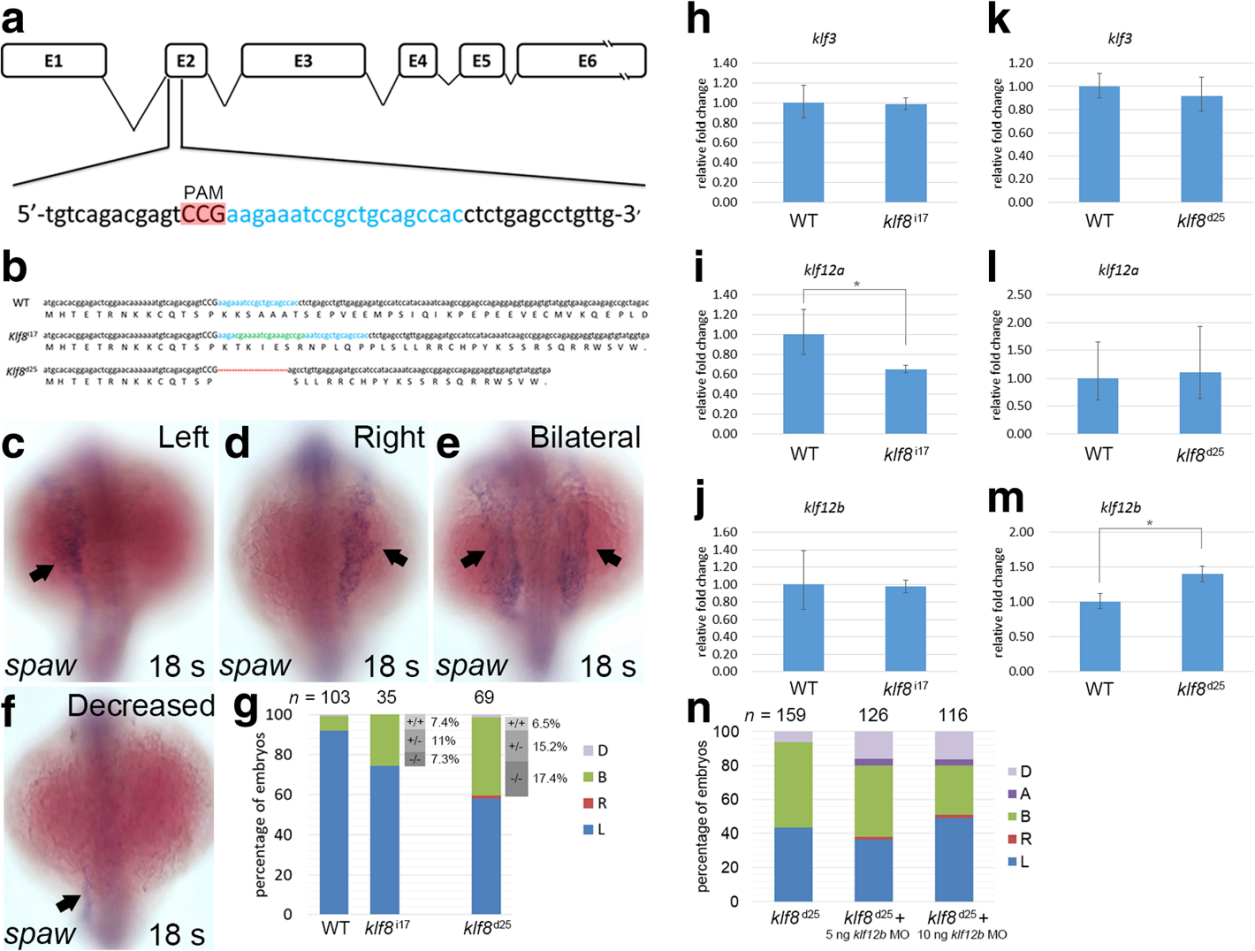
Generation of *klf8* mutants by CRISPR-Cas9 gene editing and the effect on *spaw* expression. **a** *klf8* genomic structure with *klf8* sgRNA (*blue lettering*) targeted to exon 2. Protospacer adjacent motif (PAM) sequence is shown in *red*. **b** Nucleotide and predicted amino acid sequences of *klf8* in wild type, *klf8* ^I17^ and *klf8d25* mutants are shown. Deleted nucleotides are shown by a *red dashed line*, while inserted nucleotides are shown in *green lettering*. Representative images of embryos with different *spaw* expression patterns in the LPM at 18 s stage (*arrow*; **c-f**). **g** Percentage of embryos displayed left (L), right (R), decreased (D) or bilateral (B) expression of *spaw* in the LPM from intercross of respective *klf8* ^*d25*^ or *klf8* ^*i17*^ F2 heterozygous mutants. Deduced percentage of wild type (+/+), heterozygote (+/−) or homozygote (−/−) genotype of embryos from intercross of respective *klf8* ^*d25*^ or *klf8* ^*i17*^ F2 heterozygous mutants exhibited bilateral *spaw* expression pattern. Expression levels of *klf3* (**h, k**), *klf12a* (**i, l**) or *klf12b* (**j, m**) were compared between wild type and respective *klf8* ^*d25*^ and *klf8* ^*i17*^ F5 homozygous mutant embryos at 10–12 s stages (**h-m**). Knockdown of *klf12b* reduced the percentage of embryos with bilateral *spaw* expression in the LPM of *klf8* ^*d25*^ F6 homozygous mutant embryos, but the reduction did not reach significance (*p* = 0.45 for the comparison between *klf8* ^*d25*^ and *klf8* ^*d25*^+ 5 ng *klf812b* MO, *p* = 0.05 for the comparison between *klf8* ^*d25*^ and *klf8* ^*d25*^+ 10 ng *klf812b* MO) (**n**). Statistical significance was determined by Student’s *t*-test. * *p* < 0.05. *Error bars* indicate standard deviation

In order to confirm our morphant results, we generated *klf8* mutants using a CRISPR-Cas9 system. Although we designed three *klf8* sgRNAs targeting to exon 2 or exon 3, only *klf8* sgRNA1, which targets to exon 2, was successful in producing mutants. Administration of sgRNA1 induced efficient deletion or insertion of bases in exon 2 and resulted in two *klf8* mutant alleles (*klf8* ^*d25*^ and *klf8* ^*i17*^) (Fig. 6a). The *klf8* ^*d25*^ mutant had a 25 bp deletion, which produced a 35 amino acid-long misframed Klf8 protein, while the *klf8* ^*i17*^ mutant had a 17 bp insertion that generated a 49 amino acid-long misframed Klf8 protein (Fig. 6b).

Of note, in 24 hpf homozygous F3 embryos of *klf8* ^*d25*^ and *klf8* ^*i17*^ mutants, we did not observe smaller eyes and abnormal cerebellar morphology that were detected in *klf8* morphant embryos [35]. Next, we investigated whether *spaw* expression was affected in *klf8* mutant embryos. Bilateral *spaw* expression was more frequently observed in embryos from the intercross of respective *klf8* ^*d25*^ (27 out of 69, 39.1%) or *klf8* ^*i17*^ (9 out of 35, 25.7%) F2 heterozygous mutants as compared to wild type embryos (7%) at 18 s stage (Fig. 6c-g). In order to evaluate the genotype of F3 embryos from *klf8* ^*i17*^ and *klf8* ^*d25*^ mutants with bilateral *spaw* expression, we sequenced seven *klf8* ^*i17*^ and 18 *klf8* ^*d25*^embryos with the phenotype. From sequencing data, we obtained two (28.6%) wild type, three (42.9%) heterozygotes and two (28.5%) homozygotes from a total of seven *klf8* ^*i17*^ F3 embryos, as well as three (16.7%) wild type, seven (38.9%) heterozygotes and eight (44.4%) homozygotes from a total of 18 *klf8* ^*d25*^ embryos. We then deduced that 7.3% of *klf8* ^*i17*^ or 17.4% of *klf8* ^*d25*^ F3 embryos were homozygous mutant embryos that also had bilateral *spaw* expression. This observation was based on the following calculation [0.285 (% of sequenced embryos that were homozygous) × 9 (total number with bilateral *spaw* expression) / 35 (total number of embryos) = 7.3% for *klf8* ^*i17*^; 0.444 × 27 / 69 = 17.4% for *klf8* ^*d25*^]. The other F3 embryos that exhibited bilateral *spaw* expression were also deduced to be either heterozygous mutant embryos (11% in *klf8* ^*i17*^, 15.2% in *klf8* ^*d25*^) or sibling wild type (7.4% in *klf8* ^*i17*^, 6.5% in *klf8* ^*d25*^) based on similar calculations.

Because human KLF8, KLF3 and KLF12 form a subgroup in phylogenetic tree analysis due to the presence of CtBP-binding sites [41], we wondered whether expression of zebrafish *klf3*, *klf12a*, or *klf12b* may be upregulated to compensate for *klf8* deficiency. We discovered substantial upregulation of *klf12b* in *klf8* ^*d25*^ F5 homozygous mutant embryos and downregulation of *klf12a* in *klf8* ^*i17*^ F5 homozygous mutant embryos at 10–12 s stages, while no alteration of *klf3* expression was observed in either *klf8* ^*d25*^ or *klf8* ^*i17*^ mutant embryos (Fig. 6h-m). Subsequently, we knocked down *klf12b* in *klf8* ^*d25*^ F6 homozygous mutant embryos and evaluated the *spaw* expression pattern at 18 s stage (Fig. 6n). In *klf8* ^*d25*^ F6 homozygous mutant embryos, bilateral (50.3%) and decreased (6.3%) *spaw* expression patterns were detected. In homozygous mutant embryos injected with 5 or 10 ng *klf12b* MO, we found a dose-dependent reduction that did not reach significance in the percentage (42.1% for 5 ng, 29.3% for 10 ng) of embryos with bilateral *spaw* expression, which was accompanied by the increased occurrence of right (1.6% for 5 ng, 1.7% for 10 ng), decreased (15.9% for 5 ng, 16.4% for 10 ng) or absent (4.0% for 5 ng, 3.4% for 10 ng) *spaw* expression patterns.

These results indicate that although *klf8* mutant embryos display different *spaw* expression patterns than morphant embryos, this effect may be partly attributed to a compensatory induction of *klf12b* expression.

## Discussion

Establishing asymmetric *spaw* expression in the LPM is essential for left-right patterning in zebrafish embryos. Expression of *spaw* is first apparent in bilateral cells flanking KV between the 4–6 s stages, while asymmetric *spaw* expression emerges in the posterior LPM during the 10–12 s stages, and extends to the anterior LPM by the 18 s stage [22]. Knockdown of *spaw* abolishes *spaw* expression in the LPM but not in peri-KV domains [21], suggesting that autoregulation of *spaw* occurs only in the left LPM. We used morpholino antisense oligomers to knockdown *klf8*, and found that *spaw* expression in the left LPM was reduced or eliminated in the majority of 18–22 s stage morphants (Fig. 2, Additional file 2: Figure S2). Spaw activates expression of itself, as well as *lft1*, *lft2*, and *pitx2* in the left LPM during the segmentation stage [22]. As such, the observed reduction or elimination of *lft1*, *lft2*, and *pitx2* in the left diencephalon, heart or LPM at 18–22 s stages in the majority of *klf8* morphants was not unexpected (Fig. 2, Additional file 2: Figure S2). Overall, defects in the expression of *spaw* and its downstream genes, and the subsequent defects in internal organ patterning observed in *klf8* morphants are consistent with those detected in *spaw* morphants [22]. Although *spaw*/*sfw* mutant embryos more frequently displayed a D-loop heart (68%), compared to *klf8* morphants, the difference may be attributed to heart specific actomyosin activity. Furthermore, in *sfw* mutant embryos, expression of laterality genes including *lft1*, *lft2*, and *pitx2* were lost, and *spaw* expression did not propagate toward the anterior of the left LPM [42]. In our study, we saw that overexpression of *klf8* but not *klf8△zf* resulted in bilateral expression of *spaw*, *lft1*, *lft2*, and *pitx2* at 18 s or 19–22 s stages (Fig. 4, Additional file 3: Figure S3), demonstrating a requirement for the Klf8 zinc finger DNA binding domain. Expression of *ntl* in the notochord was found to be unaltered in *klf8*-overexpressing embryos, suggesting that the midline structure is in intact. Overall, our results demonstrate that Klf8 may regulate asymmetric *spaw* expression in the left LPM, which in turn affects left-side specific expression of *lft1*, *lft2*, and *pitx2* in zebrafish embryos.

Proper morphogenesis of KV is also important for initial asymmetric *spaw* expression in the posterior LPM. The development of KV involves the formation of DFCs from surface epithelial cells, ingression at the dorsal germ ring margin, DFC migration, formation of rosette-like epithelial structures, coalescence of epithelial rosettes, and differentiation of ciliated KV with interior lumen [15, 43]. Both Tbx16 and Ntl were shown to regulate a mesenchymal to epithelial transition that participates in the formation of rosette-like epithelia [44]. Wnt11- and Prickle1a-mediated planar cell polarity signalling, as well as Cnpy1-mediated FGF signalling, were shown to regulate cell adhesion between adjacent dorsal forerunner cells to maintain cluster formation [45, 46]. Defects in these signalling events resulted in small KV lumen, with shortened and decreased number of KV cilia. In *klf8* morphants, a similar smaller KV lumen, with decreased number and length of KV cilia was frequently observed at the 10s stage. These abnormal structures may result from lower number of DFCs that was observed in 75% epiboly morphants (Fig. 5). Whether Klf8 may participate in Wnt11- and Prickle1a-mediated planar cell polarity signalling, or Cnpy1-mediated FGF signalling to modulate DFC cluster formation, remains to be determined.

Zebrafish KV architecture is asymmetric along the anterior-posterior axis, with more ciliated cells in the anterior region. Furthermore, the positioning of the basal body of motile cilia at the posterior end of the epithelial cells may result in cilia tilting [17, 47]. These motile cilia then use a vortical motion to generate swirling fluid flow consisting of a relatively stronger leftward flow across the anterior pole of KV and a weaker rightward flow at the posterior end [16, 18]. Based on experimental tracking of native particles within the KV of wild type, *did* ^−/−^ mutant and *dnah7* morphants, and simulated flow by mathematically modelling, it was determined that a threshold of 30 cilia, with dorsal anterior clustering, is essential to generate proper swirling flow in the anti-clockwise direction [40]. In control embryos, with strong left-sided flow across the anterior pole of KV, asymmetric expression is established for *charon* in the right side of the KV, and *spaw* in the left LPM. In embryos with non-directional flow, symmetric *charon* expression and a lack of *spaw* expression may be found. Embryos without motile cilia, and therefore no KV flow, may exhibit symmetric and slightly weaker *charon* expression and bilateral *spaw* expression in the posterior LPM [40]. In *klf8* morphant embryos, injected with different *klf8* MOs, a significantly reduced number of cilia (< 30), with random distribution was detected (Fig. 5). KV with such a cellular architecture may exhibit a weak and homogenous fluid flow, resulting in the symmetric *charon* expression around KV that was detected in the majority of *klf8* morphants, and leading to drastically reduced *spaw* expression at late somite stage (Additional file 4: Figure S4, Fig. 2). Overall, our results clearly indicate that Klf8 is required for normal KV morphogenesis, which is known to be critical for initiating asymmetric *spaw* expression in the left LPM.

In mouse and chick, BMP signalling plays either a positive or negative role in regulating asymmetric *Nodal* expression. Moreover, the presence of BMP antagonists, such as Noggin, Chordin, or Caronte, can relieve BMP-inhibition to promote asymmetric *Nodal* expression in the left LPM [48–51]. In zebrafish embryos, heat–activated *BMP2b* expression inhibits *spaw* expression, while heat-activated *noggin3* induced bilateral *spaw* expression, indicating that BMP signalling is required to repress *spaw* expression in the right LPM of early segmentation stage embryos, [25]. In addition, expression of Lft1 in the midline, and Lft2 in the left cardiac field, serve to generate a posterior or anterior barrier to restrict Spaw activity to the left LPM during segmentation stages in zebrafish embryos [27]. Dvr1, a zebrafish Vg1 ortholog, was also shown to facilitate the transfer of *spaw* expression from the peri-KV region to the left LPM. Thus, reduced or absent expression of *spaw* and downstream *lft1* and *lft2* in the LPM, diencephalon, notochord, or heart were detected in *dvr1* morphants [52]. In order to investigate whether Klf8 may regulate asymmetric *spaw* expression via modulation of expressions of *bmp2b* or *dvr1*, we then compared expression of these two genes between *klf8* morphants and control embryos (Additional file 5: Figure S5). Similar *bmp2b* expression level around tailbud region was identified in 3 s stage wild type and embryos injected with *klf8*-MO1^atg^, *klf8*-MO2^atg^ or *klf8*- 4 mm MO1. Likewise, no alteration of *dvr1* expression around the tailbud region was detected in wild type and embryos injected with *klf8*-MO1^atg^, *klf8*-MO2^atg^ or *klf8*-4 mm MO1 at bud stage. Thus, Klf8 does not act via BMP2b or Dvr1 signalling pathway to regulate asymmetric *spaw* expression, and the underlying mechanism remains to be determined.

In addition to our studies with *klf8* morphants, we generated *klf8* mutants by a CRISPR-Cas9 system (Fig. 6). Intriguingly, obvious phenotypic differences were found between morphants and mutants. In 24 hpf homozygous F3 embryos of *klf8* ^*d25*^ and *klf8* ^*i17*^ mutants, we did not observe smaller eyes and abnormal cerebellar morphology that were detected in *klf8* morphant embryos [35]. In addition, bilateral *spaw* expression was detected in the LPM of *klf8* ^*d25*^ and *klf8* ^*i17*^ mutants at the 18 s stage (Fig. 6). Discrepant phenotypes between mutants created by TALENs or CRISPR-Cas genome editing systems and antisense morpholino mediated-morphants have been frequently encountered. Previously, differences have been attributed to off-target effects of morpholinos [53], or compensatory effects, which have been described in vasculature development [54], reproduction [55], or neurogenesis [56]. With regard to the two *klf8* mutant alleles from our study, more *klf8* ^*d25*^ mutant embryos showed bilateral *spaw* expression in the LPM, compared to *klf8* ^*i17*^ mutants (Fig. 6g, n). This difference in outcome may correlate with aberrant upregulation of *klf12b* in *klf8* ^*d25*^ that was further confirmed by *klf12b* knockdown, but not in *klf8* ^*i17*^, mutants (Fig. 6n). Similar compensatory inconsistency was found in *stmn4* mutants, which had a low (< 10%) portion of embryos showing similar phenotype to *stmn4* morphants. Interestingly, the authors found that *stmn1b* was upregulated to compensate in *stmn4△5* but not *stmn4△4* mutants [56]. In our study, we observed that in response to *klf8* deficiency, *klf12b*, a member of a subgroup of KLF family with a CtBP interaction site, was induced to compensate for the loss of *klf8*. However aberrant upregulation of *klf12b* further resulted in bilateral *spaw* expression. On the contrary, downregulation of *klf12a* was detected in *klf8* ^*i17*^ mutants (Fig. 6). In these mutants, bilateral *spaw* expression was observed to a lesser degree, suggesting that *klf12a* may have undergone functional divergence with *klf12b*, and as such, *klf12a* may play a role in restricting *spaw* expression to the left side of embryos. Overall, *klf8* mutant embryos showed bilateral *spaw* expression, which was quite different from *klf8* morphants that exhibited reduced or eliminated *spaw* expression in the LPM. This dissimilar phenotype may have been partly related to the compensatory induction of *klf12b* expression in the mutant embryos.

## Conclusions

In this report, we have demonstrated a novel role for zebrafish Klf8 in left-right asymmetric patterning. During gastrulation, Klf8 may regulate DFC cell number to control proper KV morphogenesis, which is essential to initiate asymmetric *spaw* expression in the left LPM. During somitogenesis, Klf8 may further modulate asymmetric *spaw* expression in the left LPM to ensue asymmetric organ positioning.

## Additional files

Additional file 1: Figure S1. Knockdown of *klf8* expression by splicing morpholino oligomers resulted in embryos with reduced or absent *spaw* expression in the left LPM. **A** *klf8* genomic structure showing position of translational morpholino oligomers (*klf8*-MO1^atg^, *klf8*-MO2^atg^) and splicing morpholino oligomers (*klf8* ^DO2^, *klf8* ^AC^, *klf8* ^DO^). Arrows indicate the positions of forward and reverse primers. **B** RT-PCR showing the efficacy of *klf8* splicing morpholino oligomers. **C** The majority of embryos injected with *klf8* splicing MOs had decreased or absent *spaw* expression in the left LPM. (TIFF 153 kb)

Additional file 2: Figure S2. Heart looping and downregulated expression of *spaw* and its downstream genes were not caused by induction of *p53* expression in *klf8* morphants. Embryos co-injected with p*53*-MO^sp^ and *klf8*-MO1^atg^ or *klf8*-MO2^atg^ displayed no-loop or L-loop heart defects at 72 hpf (**A**). The majority of embryos co-injected with p*53*-MO^sp^ and *klf8*-MO1^atg^ or *klf8*-MO2^atg^ exhibited decreased or absent expression of *spaw* (**B**), *lft1* (**C**), *lft2* (**D**), or *pitx2* (**E**) in the left LPM, diencephalon or heart at the 18–22 s stages. Expression levels of *gata5* and *oep* which are known to be expressed in the LPM at the 22 s stage were unaffected by *klf8* knockdown (**F-M**). (TIFF 797 kb)

Additional file 3: Figure S3. Overexpression of *klf8* but not *klf8△zf* mRNA induced bilateral expression of Nodal signalling component genes. Percentages of embryos injected with either 100 pg of *klf8* or *klf8△zf* mRNA that exhibit left (L), right (R), decreased (D) or bilateral (B) expression of *spaw* in the LPM, *lft1* in the diencephalon and heart, *lft2* in the heart, and *pitx2* in the LPM at 18 s or 19–22 s stages. (TIFF 74 kb)

Additional file 4: Figure S4. Symmetric *charon* expression around KV was observed in the majority of *klf8* morphants. Representative images of embryos showing stronger *charon* expression on the right side (**A**) or left side (**B**) and symmetric *charon* expression on both sides (**C**) of KV are shown. Quantification of different *charon* expression patterns in embryos injected with different *klf8* MOs, control MO or wild type embryo is shown (**D**). Statistical significance was determined by Student’s *t*-test. * *p* < 0.05. Error bars indicate standard deviation. (TIFF 397 kb)

Additional file 5: Figure S5. Expression level of *bmp2b* or *dvr1* around tailbud region was not affected by *klf8* knockdown. Representative images show similar expression level of *bmp2b* (**A-D**) or *dvr1* (**E-H**) around the tailbud region in the wild type or embryos injected with *klf8*-MO1^atg^, *klf8*-MO2^atg^ or *klf8*-4 mm MO1 at 3 s or bud stages. (TIFF 382 kb)

## Abbreviations

ASE: Left-side specific enhancers
CRISPR-Cas: Clustered regularly interspaced short palindromic repeats- CRISPR-associated system
CtBP: C-terminal binding protein
DFCs: Dorsal forerunner cells
Hpf: Hours post fertilization
klf8: krüppel-like factor 8
KV: Kupffer’s vesicle
lft1: lefty1
lft2: lefty2
LPM: Lateral plate mesoderm
MO: Morpholino oligomer
Ndr: Nodal-related genes
RT: Room temperature
RT-PCR: Reverse transcription PCR
RT-qPCR: Reverse transcription quantitative real-time PCR
S: Somite
