# Supplementary figures and images for: Klf8 regulates left-right asymmetric patterning through modulation of Kupffer’s vesicle morphogenesis and spaw expression
Source: J Biomed Sci. 2017 Jul 17;24:45. doi: 10.1186/s12929-017-0351-y (PMC5513281; doi:10.1186/s12929-017-0351-y)

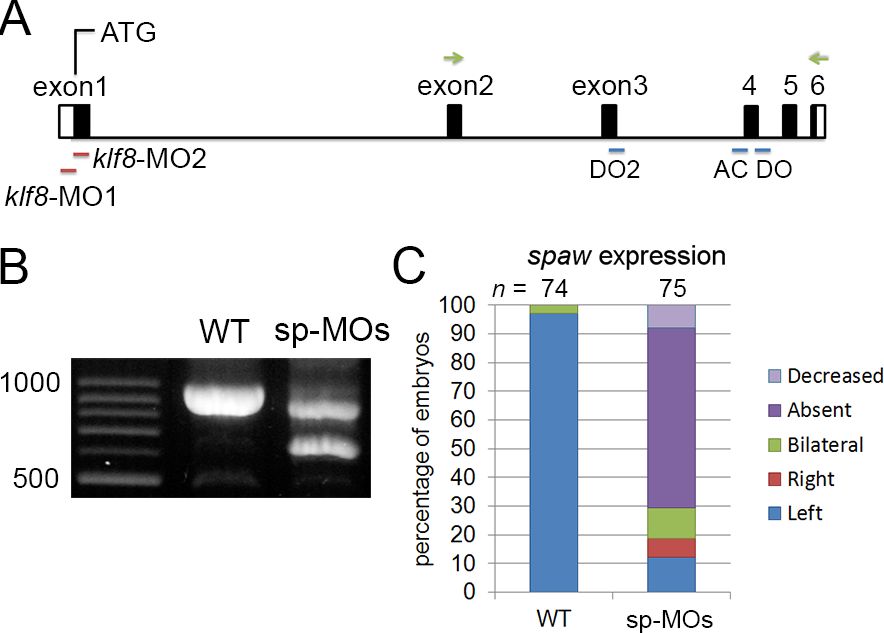

Supplement: Supplementary file 1 — Knockdown of klf8 expression by splicing morpholino oligomers resulted in embryos with reduced or absent spaw expression in the left LPM. A klf8 genomic structure showing position of translational morpholino oligomers (klf8-MO1atg, klf8-MO2atg) and splicing morpholino oligomers (klf8 DO2, klf8 AC, klf8 DO). Arrows indicate the positions of forward and reverse primers. B RT-PCR showing the efficacy of klf8 splicing morpholino oligomers. C The majority of embryos injected with klf8 splicing MOs had decreased or absent spaw expression in the left LPM. (TIFF 153 kb) [file 12929_2017_351_MOESM1_ESM.tiff]

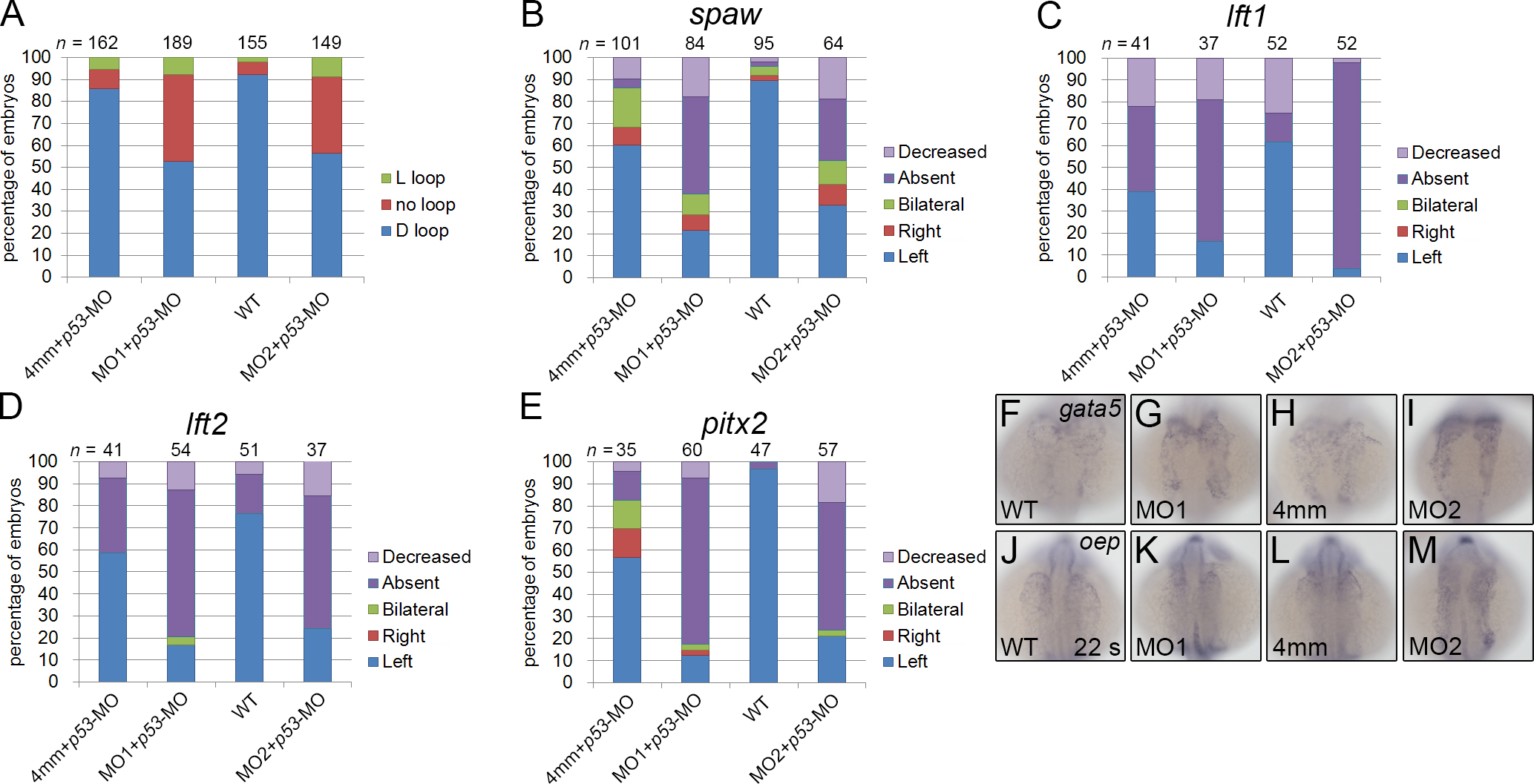

Supplement: Supplementary file 2 — Heart looping and downregulated expression of spaw and its downstream genes were not caused by induction of p53 expression in klf8 morphants. Embryos co-injected with p53-MOsp and klf8-MO1atg or klf8-MO2atg displayed no-loop or L-loop heart defects at 72 hpf (A). The majority of embryos co-injected with p53-MOsp and klf8-MO1atg or klf8-MO2atg exhibited decreased or absent expression of spaw (B), lft1 (C), lft2 (D), or pitx2 (E) in the left LPM, diencephalon or heart at the 18–22 s stages. Expression levels of gata5 and oep which are known to be expressed in the LPM at the 22 s stage were unaffected by klf8 knockdown (F-M). (TIFF 797 kb) [file 12929_2017_351_MOESM2_ESM.tif]

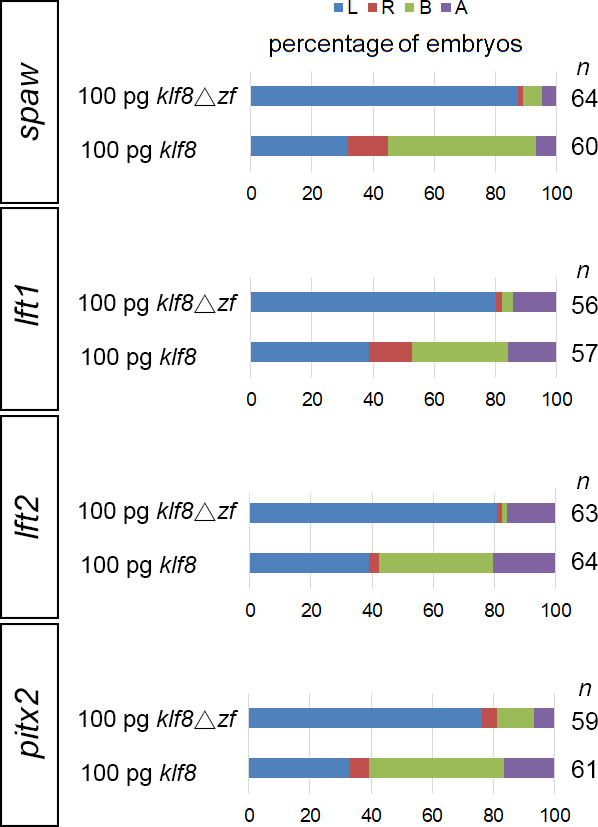

Supplement: Supplementary file 3 — Overexpression of klf8 but not klf8△zf mRNA induced bilateral expression of Nodal signalling component genes. Percentages of embryos injected with either 100 pg of klf8 or klf8△zf mRNA that exhibit left (L), right (R), decreased (D) or bilateral (B) expression of spaw in the LPM, lft1 in the diencephalon and heart, lft2 in the heart, and pitx2 in the LPM at 18 s or 19–22 s stages. (TIFF 74 kb) [file 12929_2017_351_MOESM3_ESM.tiff]

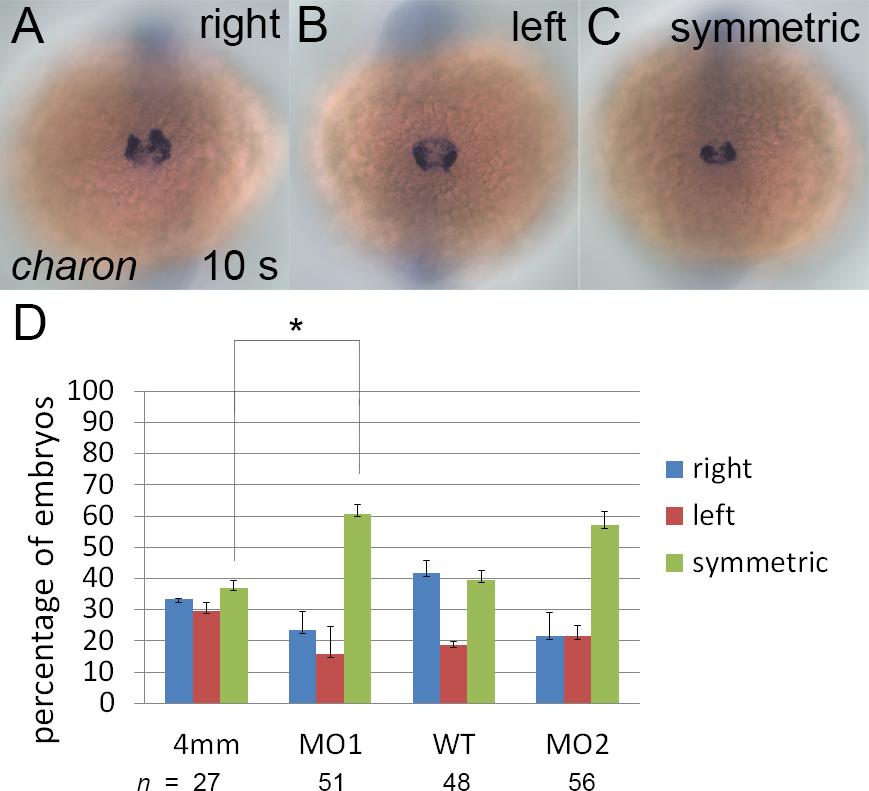

Supplement: Supplementary file 4 — Symmetric charon expression around KV was observed in the majority of klf8 morphants. Representative images of embryos showing stronger charon expression on the right side (A) or left side (B) and symmetric charon expression on both sides (C) of KV are shown. Quantification of different charon expression patterns in embryos injected with different klf8 MOs, control MO or wild type embryo is shown (D). Statistical significance was determined by Student’s t-test. * p < 0.05. Error bars indicate standard deviation. (TIFF 397 kb) [file 12929_2017_351_MOESM4_ESM.tif]

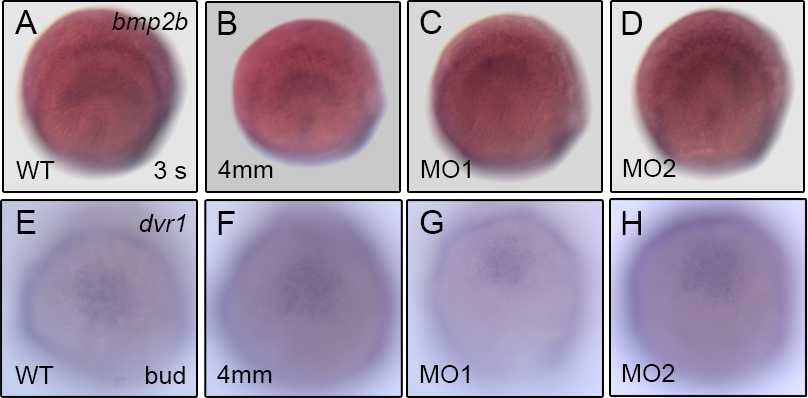

Supplement: Supplementary file 5 — Expression level of bmp2b or dvr1 around tailbud region was not affected by klf8 knockdown. Representative images show similar expression level of bmp2b (A-D) or dvr1 (E-H) around the tailbud region in the wild type or embryos injected with klf8-MO1atg, klf8-MO2atg or klf8-4 mm MO1 at 3 s or bud stages. (TIFF 382 kb) [file 12929_2017_351_MOESM5_ESM.tiff]
